# Pneumolacrimia: A Proposed Terminology and Classification System for Nasolacrimal Drainage System Aeration on Computed Tomography

**DOI:** 10.7759/cureus.115328

**Published:** 2026-08-27

**Authors:** Abharna Arun, Eileen B Kuriakose, Lekhya Venkataraman, Aathithiya P Balaji, Kamal Raman, Thirunavukarasu Palanisamy

**Affiliations:** 1 Department of ENT-Head and Neck Surgery, Sri Ramachandra Institute of Higher Education and Research, Chennai, IND

**Keywords:** anatomical variants, computed tomography, concha bullosa, lacrimal sac aeration, nasolacrimal drainage system, paranasal sinuses, pneumolacrimia, radiological classification, sinonasal pneumatization, valve insufficiency

## Abstract

Introduction: Aeration of the nasolacrimal drainage system (NLDS) is an uncommon anatomical variant observed on computed tomography (CT) scans of the paranasal sinuses. Despite incidental identification, its clinical relevance remains unclear, and current literature is notably sparse, with only two publications on the topic; both authored by the same researcher and published outside otorhinolaryngology journals. The primary objective of this study is to characterize the radiological features of the nasolacrimal apparatus on CT, determine the prevalence of nasolacrimal pneumatization, and propose a standardized terminology and classification system, termed "pneumolacrimia." As a secondary, descriptive objective, we report the co-occurrence of nasolacrimal pneumatization with other sinonasal pneumatization patterns within the affected cohort, and propose a hypothesis, requiring further anatomical or functional validation, regarding a possible underlying mechanism

Materials and methods: This retrospective study included 250 subjects (500 nasolacrimal apparatuses ) who underwent paranasal sinus CT imaging at a tertiary care hospital between 2023 and 2025, retrieved from the institutional Picture Archiving and Communication System (PACS). Prevalence is side-based. Cases showing air within the nasolacrimal apparatus were categorized as type I (partial aeration, <50% of vertical height) or type II (complete aeration, ≥50%). Associated paranasal sinus pneumatization patterns and anatomical variations were documented. Statistical analysis assessed correlations with age, gender, and other sinus variants.

Results: Pneumolacrimia (air in the NLDS) was present in 27 (11%) cases, more commonly with complete aeration. No significant gender differences were found. The highest prevalence occurred in the 31-40-year age group. Concha bullosa was present in 30 (73.2%) of cases with NLDS aeration, 20 (66.67%) of which were ipsilateral. Sphenoid pneumatization most commonly followed type II pattern (21, 51.2%), while frontal sinuses were most often hyperplastic (24, 58.5%).

Conclusion: NLDS aeration, though rare, is a recognizable entity on CT and may reflect valvular insufficiency, agenesis, or dysfunction. We propose the term pneumolacrimia to describe this radiological finding and introduce a two-tier classification to aid in standardized reporting. Further anatomical and multicentric studies are needed to validate these findings and explore their clinical implications.

## Introduction

The main route for tear drainage from the conjunctival sac to the nasal cavity is the nasolacrimal drainage system (NLDS). The various parts of the nasolacrimal apparatus form a continuous drainage pathway. The nasolacrimal duct (NLD), which runs in the bony nasolacrimal canal and is about 17 mm long, is the last segment of the nasolacrimal apparatus [1].

The NLDs are opaque in the majority of normal people on computed tomography (CT) imaging [2], with the bony canal formed by the lacrimal bone, maxilla, and inferior turbinate, creating a protected pathway for the membranous structures. Pneumatization of the nasolacrimal apparatus represents an uncommon anatomical variant that can be identified on CT scans of the paranasal sinuses.

Review of the literature reveals a significant paucity of data regarding the aeration of the nasolacrimal system on computed tomography. A PubMed search restricted to English-language articles, from database inception to May 2025, was conducted using the terms "aeration," "pneumatization," "nasolacrimal apparatus," and "nasolacrimal duct," individually and in combination. Only two pertinent publications were identified. This demonstrates the narrow focus of the field's present research.

This study intends to characterize the radiological features of the nasolacrimal apparatus, the incidence of aerated lacrimal sacs, categorize the various patterns of aeration and extent of aeration, and propose a standardized terminology and classification system. It should be noted that the clinical significance of an aerated nasolacrimal system remains uncertain; this study does not presume any pathological association but instead aims to characterize the radiological prevalence, patterns, and possible anatomical correlates of this variant. We propose the term “pneumolacrimia" to describe an aerated nasolacrimal system for a more accurate description and reporting. We also examine its potential relationship with other sinonasal pneumatization patterns and put forward a hypothesis to explain the underlying mechanism of lacrimal system aeration.

## Materials and methods

A retrospective analysis of 250 paranasal sinus CT scans, including 500 nasolacrimal apparatuses, was conducted. Each scan was evaluated alongside the corresponding ipsilateral paranasal sinuses. All included CT scans had originally been reported by multiple radiologists as part of routine clinical care. With a slice thickness of 0.625 mm, all scans were obtained using a standard imaging protocol on the GE Optima CT660 and were noncontrast-enhanced. All patients were positioned supine for the procedure. CT scans were randomly selected from the institutional Picture Archiving and Communication System (PACS), encompassing studies performed between 2023 and 2025. A total of 250 subjects were included, while the following exclusion criteria were applied: age below 16 years, history of facial and/or orbital trauma, known nasolacrimal pathology or associated symptoms (e.g., epiphora), presence of pathology obscuring visualization of the NLDS, and prior surgical intervention involving the paranasal sinuses or nasolacrimal apparatus.

For this study, scans were independently reviewed for nasolacrimal pneumatization by two ENT surgeons blinded to patient demographic and clinical data, with discrepancies resolved by consensus. The unit of analysis was the individual nasolacrimal system (side-based); prevalence figures are therefore reported per side (n = 500) rather than per subject (n = 250). The 50% vertical height threshold used to distinguish Type I from Type II pneumatization was selected as a pragmatic, reproducible cutoff consistent with grading conventions used for other paranasal sinus pneumatization patterns, though it has not been independently validated.

**Figure 1:**
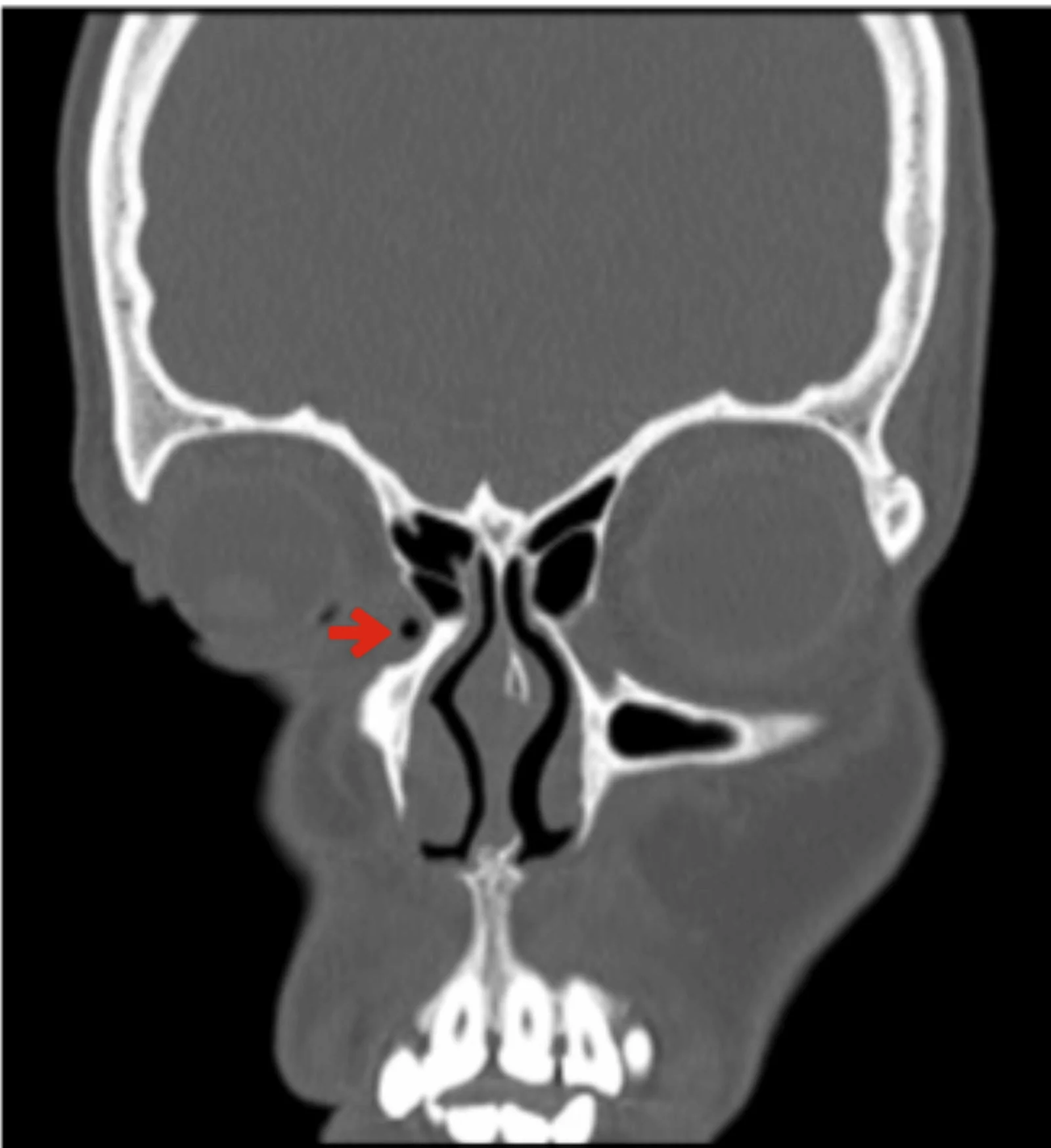
Partial pneumatization of the right lacrimal sac as indicated by the arrow

**Figure 2:**
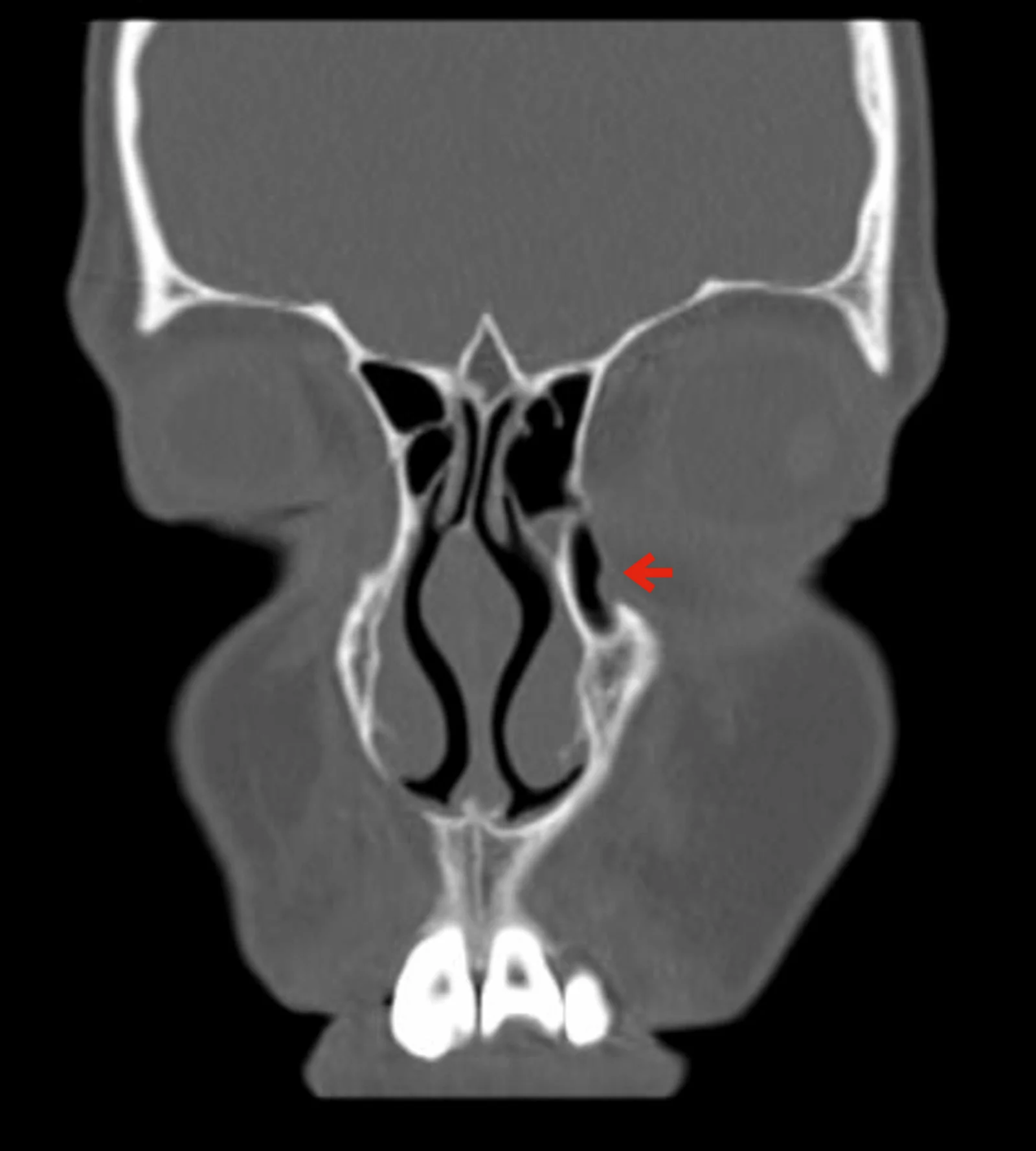
Complete pneumatization of the left lacrimal sac as indicated by arrow

Axial and coronal slices were evaluated for evidence of pneumatization within the nasolacrimal apparatus. Identified cases were classified according to the following classification system: type I (partial pneumatization), less than 50% of the vertical height of the nasolacrimal apparatus demonstrates air on coronal CT images as marked with an arrow in Figure 1; and type II (complete pneumatization): pneumatization involving 50% or more of the vertical height of the nasolacrimal apparatus on coronal CT images as shown in Figure 2.

Patterns of paranasal sinus pneumatization were analyzed to assess their association with other anatomical variants and pneumatization patterns within the nasal cavity and paranasal sinuses. Based on the classification proposed by Vaezi et al., sphenoid sinus pneumatization was categorized into three coronal types. In type I (previdian), the pneumatization extends from the midline to the medial margin of the vidian canal. In type II (prerotundum), it extends up to the lateral margin of the foramen rotundum. In type III (postrotundum), the pneumatization extends laterally to the foramen rotundum [3].

The classification of frontal sinus size was performed by the criteria established by Al Hatmi et al. This system categorizes frontal sinus development into four types. Aplasia is the complete absence of sinus pneumatization. Hypoplasia, defined by minimal pneumatization restricted below the supraorbital line. Medium-sized sinuses that demonstrate pneumatization that extends above the supraorbital line but remains medial to the mid-orbital line. Finally, hyperplasia is characterized by pneumatization that extends laterally to the mid-orbital line [4].

The statistical analysis was performed using IBM SPSS Statistics for Windows, Version 27 (Released 2019; IBM Corp., Armonk, New York, United States). Descriptive statistics were used to summarize the demographic characteristics of the study population, with categorical variables presented as frequencies and percentages and continuous variables expressed as mean ± standard deviation (SD). The chi-square test was employed to assess the association between categorical variables, specifically to determine the relationship between age groups and gender vs the presence of the right and left lacrimal sac. A p-value < 0.05 was considered statistically significant, and p < 0.01 was considered highly significant.

## Results

The demographic profile of the study participants shows that the mean age was 38.06 ± 15.7 years, with the highest representation in the 31-40-year age group (61, 24.4%), followed by the 21-30-year age group (53, 21.2%), and the 41-50-year age group (39, 15.6%). The population was evenly distributed by gender, with 124 (49.6%) males and 126 (50.4%) females, indicating a balanced sample for subsequent comparative analyses. The table depicting demographic characteristics clearly shows that the 31-40 year age category constitutes the largest portion of the population, followed by the 21-30 and 41-50 year groups, with a progressively decreasing frequency in older and younger age extremes (Table 1).

**Table 1:** Demographic characteristics of the study population

Study variables	Mean ± SD	Frequency	Percentage distribution
Age group	38.06 ± 15.7		
≤20 years		37	14.8%
21-30 years		53	21.2%
31-40 years		61	24.4%
41-50 years		39	15.6%
51-60 years		36	14.4%
61-70 years		17	6.8%
>70 years		7	2.8%
Gender			
Male		124	49.6%
Female		126	50.4%

**Figure 3:**
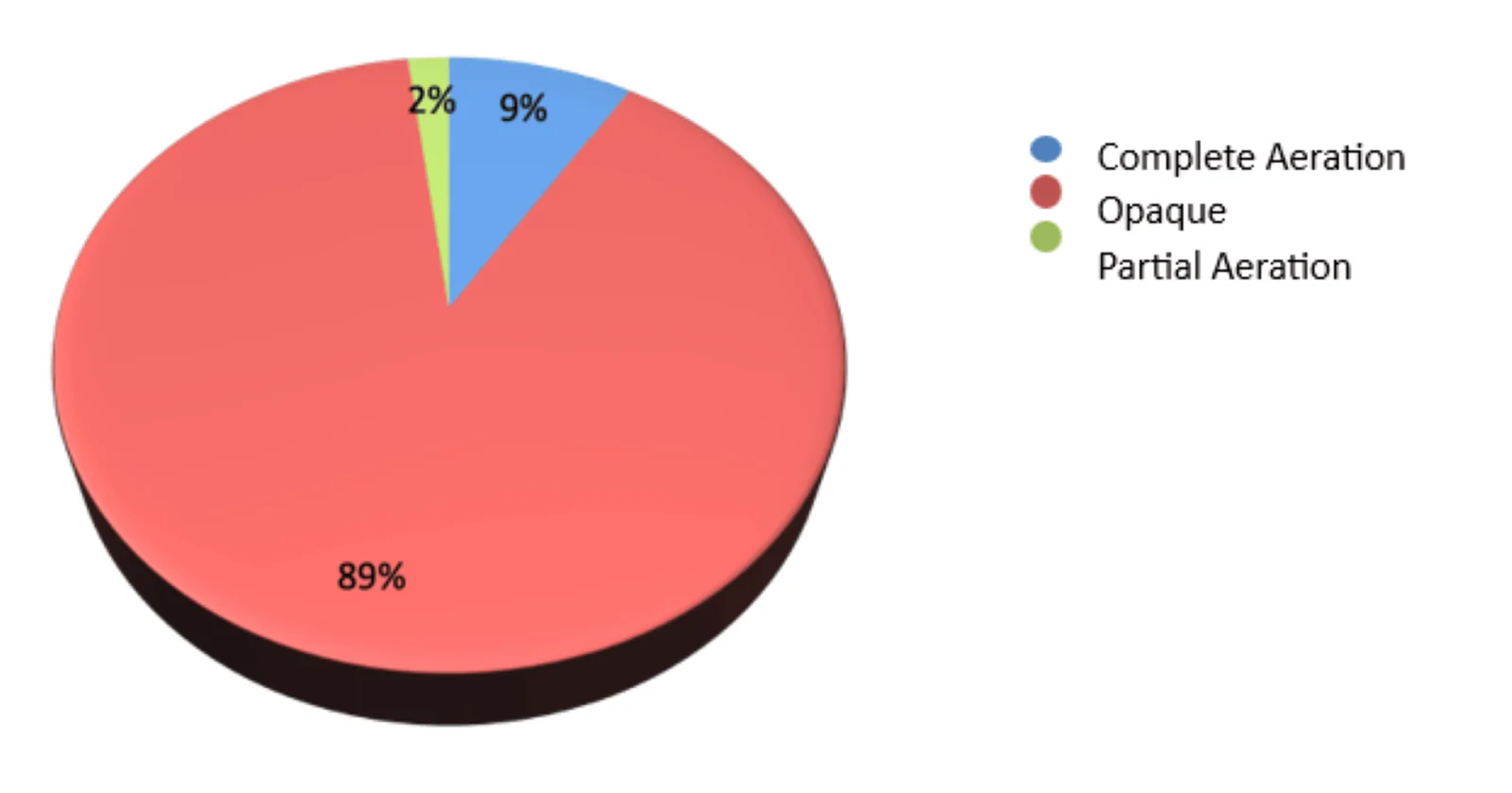
Pie chart showing the percentage distribution of overall nasolacrimal drainage system pneumatization in the study population

The anatomical assessment of the lacrimal sac revealed a significantly lower presence of pneumatization on both sides. On the right side, only 20 (8%) of cases showed the presence of complete pneumatization, and seven (2.8%) showed a partial pneumatization. On the left side, 25 (10%) showed complete pneumatization, and three (1.2%) showed partial pneumatization. The overall presence of pneumatization was noted in only 55/500 (11%) of the population (side-based), with highly significant p-values (<0.001) suggesting a notable absence of pneumatization among participants (Figure 3).

**Figure 4:**
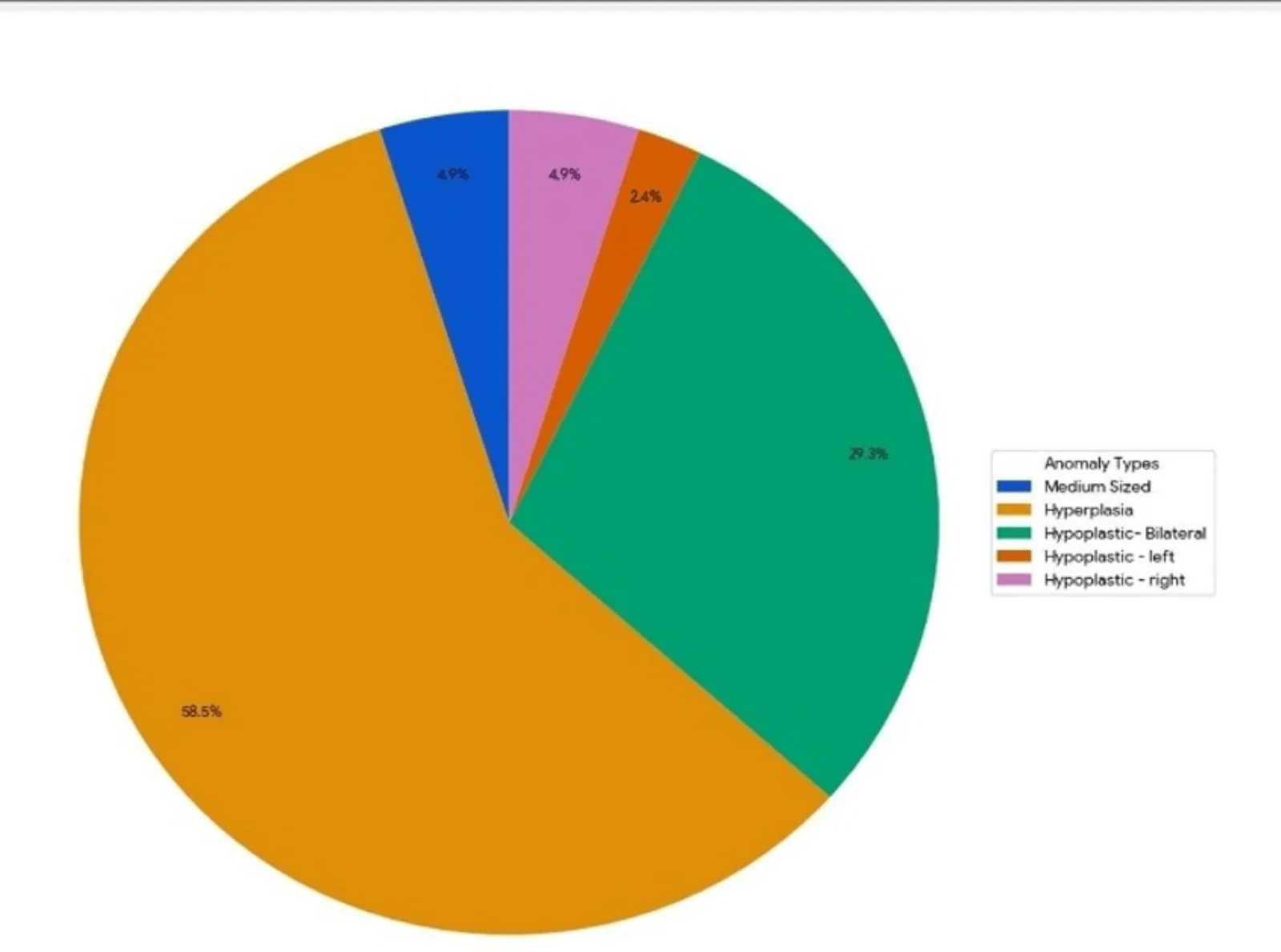
Pie chart showing the distribution of sphenoid sinus pneumatization patterns among the study population

**Figure 5:**
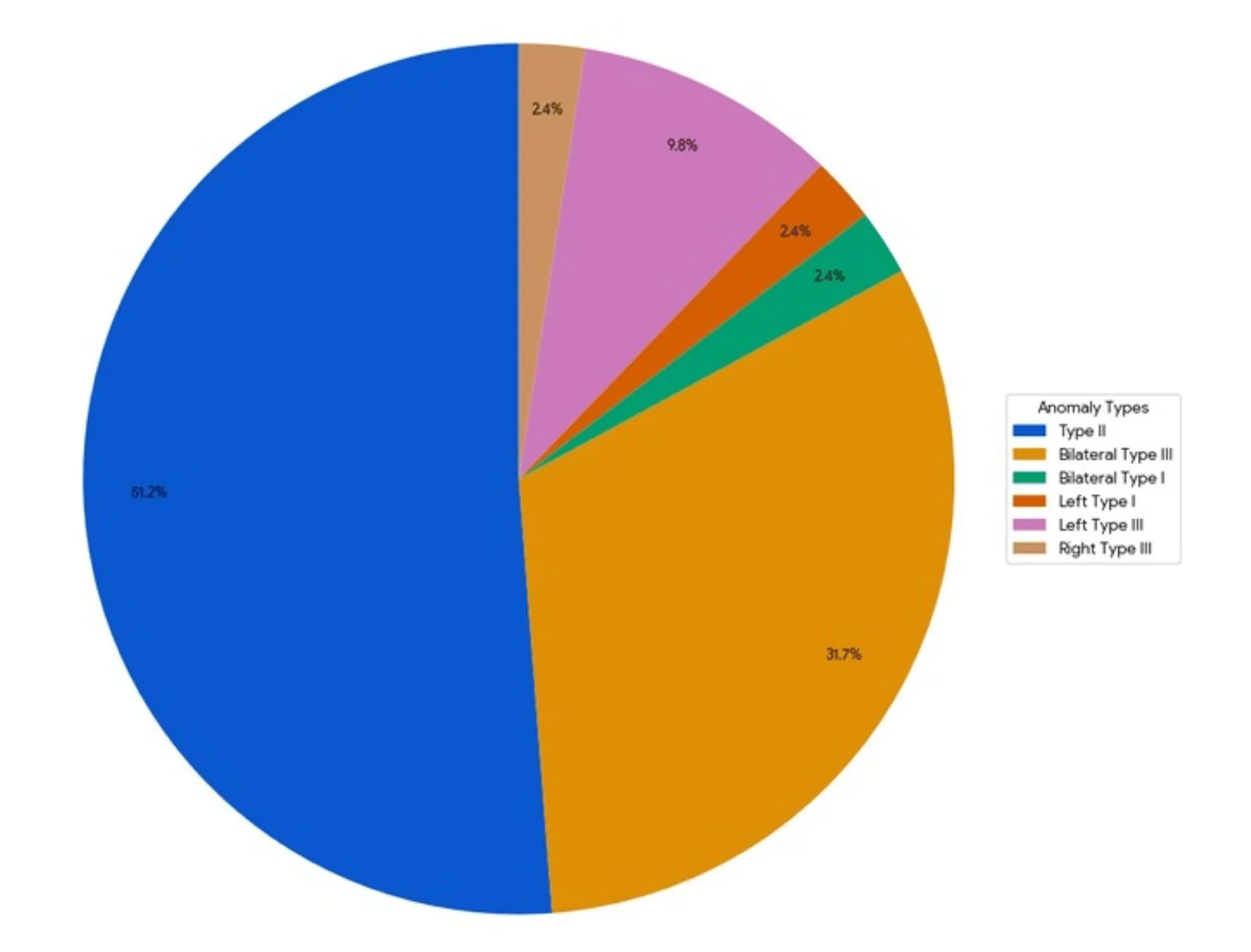
Pie chart showing the distribution of frontal sinus pneumatization patterns among the study population

When examining other anatomical pneumatization patterns, statistically significant findings were observed. Sphenoid pneumatization revealed that the most common variant was “type II pneumatization” (21, 51.2%), followed by bilateral type III (13, 31.7%), with a significant difference across patterns (p < 0.001) (Figure 4). Similarly, frontal pneumatization was significantly variable, with “hyperplastic” sinuses being the most common (24, 58.5%), followed by hypoplastic sinuses (12, 29.3%) (p < 0.001) (Figure 5). Middle turbinate anomalies, such as bilateral and unilateral concha bullosa, were seen in a total of 30 (73.2%), while 11 (26.8%) had no concha formation, showing significant variability (p = 0.001). The maxillary sinus showed predominance of “unremarkable” findings in 28 (68.3%) of subjects. Other anomalies like Onodi and Haller cells were largely absent in one and seven cases, respectively (2.4% and 17.1%, respectively) (Table 2). Anatomical variant data presented in Table 2 were recorded for the pneumolacrimia-positive subgroup (n = 41 of 43 positive subjects with complete variant data) and therefore describe the frequency of these variants within affected cases rather than a comparison against the pneumolacrimia-negative cohort.

**Table 2:** Frequency distribution of anatomical variants among pneumolacrimia-positive subjects

Variant category	n (%)	p-value*
Sphenoid pneumatization type		<0.001**
Type II	21 (51.2%)	
Bilateral type III	13 (31.7%)	
Left type III	4 (9.8%)	
Bilateral type I	1 (2.4%)	
Left type I	1 (2.4%)	
Right type III	1 (2.4%)	
Frontal sinus pneumatization		<0.001**
Hyperplasia	24 (58.5%)	
Hypoplastic-bilateral	12 (29.3%)	
Medium-sized	2 (4.9%)	
Hypoplastic-right	2 (4.9%)	
Hypoplastic-left	1 (2.4%)	
Middle turbinate		0.001**
Bilateral concha bullosa	11 (26.8%)	
Left concha	11 (26.8%)	
No concha	11 (26.8%)	
Right concha bullosa	7 (17.1%)	
Right partial concha (ascending limb)	1 (2.4%)	
Right partial concha (body) + left concha	1 (2.4%)	
Maxillary sinus		<0.001**
Unremarkable	28 (68.3%)	
Well pneumatized	11 (26.8%)	
Symmetrical-unremarkable	1 (2.4%)	
Hyperpneumatized (right)	1 (2.4%)	
Haller cells		<0.001**
Absent	40 (97.6%)	
Bilateral	1 (2.4%)	
Onodi cells		<0.001**
Absent	34 (82.9%)	
Left	4 (9.8%)	
Right	3 (7.3%)	

The rarity of Haller cells is evident, with near-complete absence, and the same is reflected for Onodi cells.

## Discussion

Air within the NLDS is not a common finding on CT scans. A prior study by Groell et al., conducted on 147 patients, reported that approximately 44 (30%) of individuals exhibited some degree of air within the NLDS [5]. In contrast, our study-based on a significantly larger sample size exclusively comprising a Southeast Asian population-found aeration in only 27 (11%) of nasolacrimal systems evaluated. This notably lower incidence may reflect differences in study population, CT technique, scan orientation, slice thickness, inclusion criteria, or observer variability between our study and that of Groell et al., rather than a true overestimation of prevalence in earlier work. Groell et al. evaluated 147 patients using an unspecified slice thickness and scan orientation, whereas our study used thin-slice (0.625 mm) supine CT in a Southeast Asian population. These technical and demographic differences may partly account for the divergent prevalence estimates, independent of any true population-level difference in nasolacrimal aeration.

We propose that aeration observed within the nasolacrimal system on CT may be attributed to valve insufficiency, valve agenesis, or even valve deficiency within the nasolacrimal drainage pathway. Under normal physiological conditions, multiple anatomical valves act as a barrier to prevent retrograde airflow from the nasal cavity toward the lacrimal sac. These include the valve of Hasner at the distal end of the NLD, the valves of Taillefer and the spiral valve of Hyrtl within the NLD, the valve of Krause near the sac-duct junction, the valve of Rosenmüller at the sac-common canaliculus junction, and the valves of Bochdalek and Foltz at the superior and inferior puncta [6].

According to Paul et al., nasolacrimal air regurgitation is an uncommon but documented complication of positive airway pressure (PAP) therapy, resulting from a failure of this multivalvular system. When these valves are insufficient, retrograde air can travel from the nasal cavity through the NLD, potentially reaching the lacrimal puncta. This insufficiency may be congenital, due to embryological defects or agenesis occurring before the eighth month of gestation, or acquired, as seen in patients who have undergone conjunctivodacryocystorhinostomy, dacryocystorhinostomy with Lester-Jones tube insertion, or silicone stent placement [7]. It is possible that similar mechanisms can be the cause of incidental aeration seen on CT in the absence of clinical symptoms, suggesting subclinical or partial valve dysfunction as a potential contributing factor. Additional cadaveric studies may be necessary to investigate and validate this hypothesis. The following mechanism is proposed as a hypothesis and has not been directly demonstrated in this study.

The existing literature has used inconsistent terminology to describe pneumatization of the nasolacrimal region, such as "partially or fully aerated" [2,8] and "fluid-filled or air-filled" [9,10]. Various terms have been employed without standardization, leading to confusion in radiological reporting and clinical communication. The need for systematic classification has become apparent as CT imaging capabilities have improved. Air within the lacrimal sac on CT may represent true anatomical pneumatization, transient retrograde air entry, an imaging artifact, or a physiological air-fluid variation; this study cannot distinguish between these possibilities, and the proposed valvular mechanism remains speculative pending anatomical or functional validation.

Based on current understanding and imaging capabilities, we propose the following standardized terminology: pneumolacrimia, as the presence of air within any portion of the NLDS visible on CT. As previously mentioned, it may be classified as type I (partial pneumatization), which is less than 50% of the vertical height of the nasolacrimal apparatus in coronal CT images; and type II (complete pneumatization), which is greater than or equal to 50% of the vertical height of the nasolacrimal apparatus in coronal CT images.

In a retrospective study involving 92 patients, Czyż et al. found that aeration of the NLDS appeared significantly more often and more completely on CT scans taken in the upright position, which highlights how patient posture can influence radiological findings [8]. In contrast, all CT scans in our study were performed with patients in supine position, allowing us to control this variable and rule out position as a factor contributing to the aeration of the nasolacrimal sac.

In a retrospective CT analysis of 92 adults, Czyz et al. demonstrated that aeration of the NLDS is significantly greater in males than females and peaks in individuals aged 30-40 years, while bony canal dimensions showed no correlation with either sex, age, or degree of aeration [11]. In contrast, our study did not show any gender bias, as the aeration rates were statistically the same for men and women. This differs from the gender-specific trend reported by Czyz et al. and may be due to differences in the study population, selection criteria, or imaging methods.

With regard to age distribution, our study identified the highest prevalence of aeration in the 31-40 year group, consistent with the findings of Czyz et al. Notably, we also observed secondary peaks in the 21-30 and 41-50 age brackets, suggesting a broader distribution of aeration across early to middle adulthood. Concha bullosa is a frequent finding on CT imaging; hence, its impact on aeration of the NLDS may be limited. A large-scale study by Kar et al., which reported a concha bullosa prevalence of 1477 (44.74%) among 3,303 subjects, may suggest that its presence may often represent a benign anatomical variant rather than a factor with significant functional implications for NLDS aeration [12]. Of the patients with aerated nasolacrimal systems in our study population, 30 (73.2%) had concha bullosa, whereas 11 (26.8%) showed no signs of conchal pneumatization. In 20 (66.67%) of patients, the concha bullosa was identified ipsilateral to the aerated NLD. This co-occurrence is descriptive and does not establish a causal or mechanistic relationship between concha bullosa and nasolacrimal pneumatization.

The lack of a functional evaluation of the nasolacrimal valve system is a study limitation. Although aeration is thought to be caused by agenesis or valve insufficiency, no physiological tests, such as lacrimal irrigation or dacryoscintigraphy, were carried out to verify valvular dysfunction. Additionally, formal interobserver agreement between the two reviewing ENT surgeons was not statistically assessed in this study; discrepant classifications were resolved by consensus. Future studies would benefit from formal agreement analysis (e.g., Cohen's kappa) to further validate the reproducibility of this classification system.

Anatomical variant data were recorded only for pneumolacrimia-positive subjects, so comparison with the negative cohort was not possible. Findings should be read as descriptive of the positive subgroup, not as evidence of association. Future studies should collect complete variant data across the full cohort for formal comparison.

Furthermore, the underlying mechanisms remain speculative because anatomical explanations, such as congenital valve defects, have been proposed but have not been substantiated by cadaveric dissection or histological correlation.

## Conclusions

Pneumolacrimia, or the presence of air within the nasolacrimal sac, represents a relatively underrecognized anatomical variant. This study provides descriptive, exploratory data on its prevalence and radiological classification; the clinical significance of an aerated nasolacrimal system remains unclear and was not assessed here. The observed co-occurrence of pneumolacrimia with other sinonasal anatomical variants is descriptive only; as comparative analysis against a pneumolacrimia-negative cohort was not performed, no association or causal relationship can be inferred from this study.

The proposed valvular insufficiency mechanism is a hypothesis and has not been functionally or anatomically validated in this study; further work involving functional testing (e.g., lacrimal irrigation, dacryoscintigraphy) or cadaveric/anatomical studies is needed to substantiate it. Larger, multicentric studies incorporating a comparison group and functional assessment are needed to validate these preliminary findings.
